# Meet the editors

**DOI:** 10.1002/ansa.202000076

**Published:** 2020-07-11

**Authors:** Lili He, Paul Trevorrow

**Affiliations:** ^1^ University of Massachusetts MA USA; ^2^ Executive Journals Editor Wiley Chichester UK


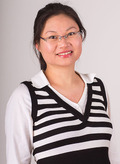


Lili He

Lili He is an associate professor of food science at the University of Massachusetts, Amherst. She received her BS and MS degrees from Zhejiang University in China and PhD degree from University of Missouri‐Columbia in the United States in 2009. Then she did her postdoc training in University of Minnesota for about 3 years. In 2012, she joined the faculty in the Food Science Department at the University of Massachusetts, Amherst as an assistant professor and in 2018 became an associate professor. Dr He's major research focus is to develop and apply the most advanced and innovative analytical techniques to help solve critical and emerging issues in food science. Her group has developed various surface‐enhanced Raman scattering (SERS) based techniques for food safety and other applications. Recent research interest has expanded to X‐ray fluorescence (XRF) spectroscopy and its applications for elemental analysis in food. Dr He's research has been strongly supported by external funding from federal and industry and published over 100 papers. In 2020, she has established the Raman, IR, and XRF Core Facility at the University of Massachusetts, Amherst. Her excellence has been recognized by receipt of the 2012 Young Scientist Award from the International Union of Food Science and Technology, 2015 American Chemical Society (ACS)‐ Agricultural and Food Chemistry Division Young Scientist Award, 2016 Young Investigator Award from Eastern Analytical Symposium, 2016 Institute of Food Technologists Samuel Cate Prescott Award for Research, and was selected as one of the Talented 12 by C&EN, the ACS magazine in 2016.

**Would you briefly explain what your research group is studying?**

Our research group is developing innovative analytical methods to solve critical and emerging issues related with food, agricultural, and environmental sciences. The main technique we focus on is surface‐enhanced Raman spectroscopy (SERS), and recently involves X‐ray fluorescence (XRF) spectroscopy too.

**Of all your research projects, which one was your favorite and why?**

My favorite project is a USDA‐NIFA funded project on studying the pesticide behaviors using SERS where we were able to analyze pesticide penetration, translocation, degradation, and persistence on and in plant tissues in situ and real‐time. We have published about 10 papers from this project and received many awards and media reports.

**What is your vision as editor on *Analytical Science Advances*?**

My vision is to establish a transparent and broadly impacted analytical community through *Analytical Science Advances* to promote the importance of analytical science and to facilitate the technical advance in various areas.

**What do you think is the key to success in a scientific career?**

Passion and efficiency.

**Who were the most influential people in your career?**

My postdoc advisor Dr. Ted Labuza who fully supported me to become an independent researcher without any hesitation.

**As a mentor and advisor, what do you advise your students in general?**

Find your career path and develop an effective methodology in your study and research.

**What do you consider to be the more exciting topics in analytical chemistry?**

Developing practically useful analytical methods to solve real problems.

**What are your views on the future of your field?**

Use an integrated approach to solve complicated problems.

**What are your favorite past‐times outside of science?**

Playing crane games, which are strategy based games with claw machines physically and on line.

**What would you do if you had 1‐year paid leave?**

Travel around the world with my family when it is safe to do so.

